# Sexual Reproduction and Seasonality of the Alaskan Red Tree Coral, *Primnoa pacifica*

**DOI:** 10.1371/journal.pone.0090893

**Published:** 2014-04-25

**Authors:** Rhian G. Waller, Robert P. Stone, Julia Johnstone, Jennifer Mondragon

**Affiliations:** 1 University of Maine, School of Marine Sciences, Darling Marine Center, Walpole, Maine, United States of America; 2 Alaska Fisheries Science Center, National Marine Fisheries Service, National Oceanic and Atmospheric Administration, Juneau, Alaska, United States of America; 3 University of Maine, Darling Marine Center, Walpole, Maine, United States of America; 4 Alaska Regional Office, National Marine Fisheries Service, National Oceanic and Atmospheric Administration, Juneau, Alaska, United States of America; Heriot-Watt University, United Kingdom

## Abstract

The red tree coral *Primnoa pacifica* is an important habitat forming octocoral in North Pacific waters. Given the prominence of this species in shelf and upper slope areas of the Gulf of Alaska where fishing disturbance can be high, it may be able to sustain healthy populations through adaptive reproductive processes. This study was designed to test this hypothesis, examining reproductive mode, seasonality and fecundity in both undamaged and simulated damaged colonies over the course of 16 months using a deepwater-emerged population in Tracy Arm Fjord. Females within the population developed asynchronously, though males showed trends of synchronicity, with production of immature spermatocysts heightened in December/January and maturation of gametes in the fall months. Periodicity of individuals varied from a single year reproductive event to some individuals taking more than the 16 months sampled to produce viable gametes. Multiple stages of gametes occurred in polyps of the same colony during most sampling periods. Mean oocyte size ranged from 50 to 200 µm in any season, and maximum oocyte size (802 µm) suggests a lecithotrophic larva. No brooding larvae were found during this study, though unfertilized oocytes were found adhered to the outside of polyps, where they are presumably fertilized. This species demonstrated size-dependent reproduction, with gametes first forming in colonies over 42-cm length, and steady oocyte sizes being achieved after reaching 80-cm in length. The average fecundity was 86 (±12) total oocytes per polyp, and 17 (±12) potential per polyp fecundity. Sub-lethal injury by removing 21–40% of colony tissue had no significant reproductive response in males or females over the course of this study, except for a corresponding loss in overall colony fecundity. The reproductive patterns and long gamete generation times observed in this study indicate that recruitment events are likely to be highly sporadic in this species increasing its vulnerability to anthropogenic disturbances.

## Introduction

Primnoidae is one of the most dominant gorgonian families in deep-sea and polar regions, with 233 valid species known to date [1]. Primnoids are found in all ocean basins and are most numerous in Antarctic waters where the family is thought to have originated [1]. This family is primarily deep-sea, with most current records coming from slope and abyssal depths whereas shallow populations are rare. The genus *Primnoa* is only found in the North Pacific, North Atlantic and subantarctic South Pacific regions, with the red tree coral, *Primnoa pacifica* Kinoshita, 1907, known only from North Pacific waters [2]. This species can attain massive size (up to 5 m in height and 7 m in width; 3, R. Stone, personal observations) and form vast monospecific thickets that provide habitat for fish [3], [4] and invertebrates [5].

Growth rates show that *P. pacifica* is a slow growing coral [6], [7] and there is an indication that successful recruitment events occur on a decadal scale (Stone pers obs). Many cold-water gorgonians appear to be gonochoristic (i.e. separate sexes at the colony level) brooders that likely have seasonal reproductive cycles [8], [9] but otherwise we know very little that will assist us in gauging their capability to recover from disturbance. Data on the reproductive processes of cold-water corals is still in its infancy compared to the wealth of data available for their shallow water zooxanthellate counterparts [10], [9], [11]. Advances in this discipline are largely hampered by inability to sample specimens at regular intervals in most deep-sea environments.

Recent submersible observations in the eastern Gulf of Alaska shelf habitats clearly indicate that red tree corals are highly vulnerable to disturbance from fishing gear and that a much higher proportion of large colonies are damaged compared to smaller colonies. Some cold-water corals subjected to mechanical disturbance exhibit reduced reproductive fitness (i.e. fecundity) via resource re-allocation and planulae expulsion [12], [13]. Furthermore, larger, more susceptible, colonies are thought to be considerably more fecund than smaller colonies [14]. Both of these factors may influence the ability of habitats dominated by red tree corals to recover from fishing gear disturbance.

Shallow-water habitats in several high-latitude fjords sustain benthic communities that are similar to those usually found in much deeper water. The term “deepwater emergence” has been used to describe this phenomenon, though the environmental factors that allow these species to survive much shallower than their usual distribution (often by thousands of meters) are not yet fully understood. Shallow-water temperatures in these fjords tend to be much cooler mimicking temperatures usually found at bathyal depths [15], [16]. Nutrient-rich upwelling, strong current regimes, temperatures at the surface layers being equal to the deep ocean and reduced light levels are other factors that could influence how these deep-sea species survive shallower than their normal distributions [7], [16]. Several shallow systems worldwide that support deepwater emerged coral species have been documented – Chile [17], Norway [18], New Zealand [19], [20], [21], Alaska [7] and British Columbia [22]. The coral species that appear in these fjord systems are often ecosystem engineers (*in sensu* [23]), altering both the local benthic and physical environment and providing suitable microhabitat for colonization of many associated species [24]. These shallow-water communities appear to mimic those of the deep sea and thus provide a unique opportunity to use the easily accessible habitats as “living laboratories” to study the physiological processes of deep-sea organisms.

A recently discovered shallow-water population of red tree corals in Tracy Arm, Holkham Bay, Southeast Alaska provided an unprecedented opportunity to collect reproductive tissue samples at regular intervals year-round from the same colonies. Red tree corals exhibit keystone species characteristics in Gulf of Alaska habitats where it provides refuge habitat for at least 12 species of rockfish (*Sebastes* sp.) including gravid females and juveniles (Stone pers obs), making it an ideal candidate for reproductive study. In September 2010, we began a 16-month study of the sexuality, reproductive mode and periodicity of *P. pacifica* from a single population within Tracy Arm Fjord. We also examined how physical damage (simulated fishing damage) might affect reproduction in this species. To our knowledge there is no other published study on any cold-water corals species that utilizes seasonal samples collected from the same individuals on a tri-monthly basis, therefore the resolution of this study is significantly higher than any reproductive information gained to date.

## Materials and Methods

### Study Site

**Figure 1:**
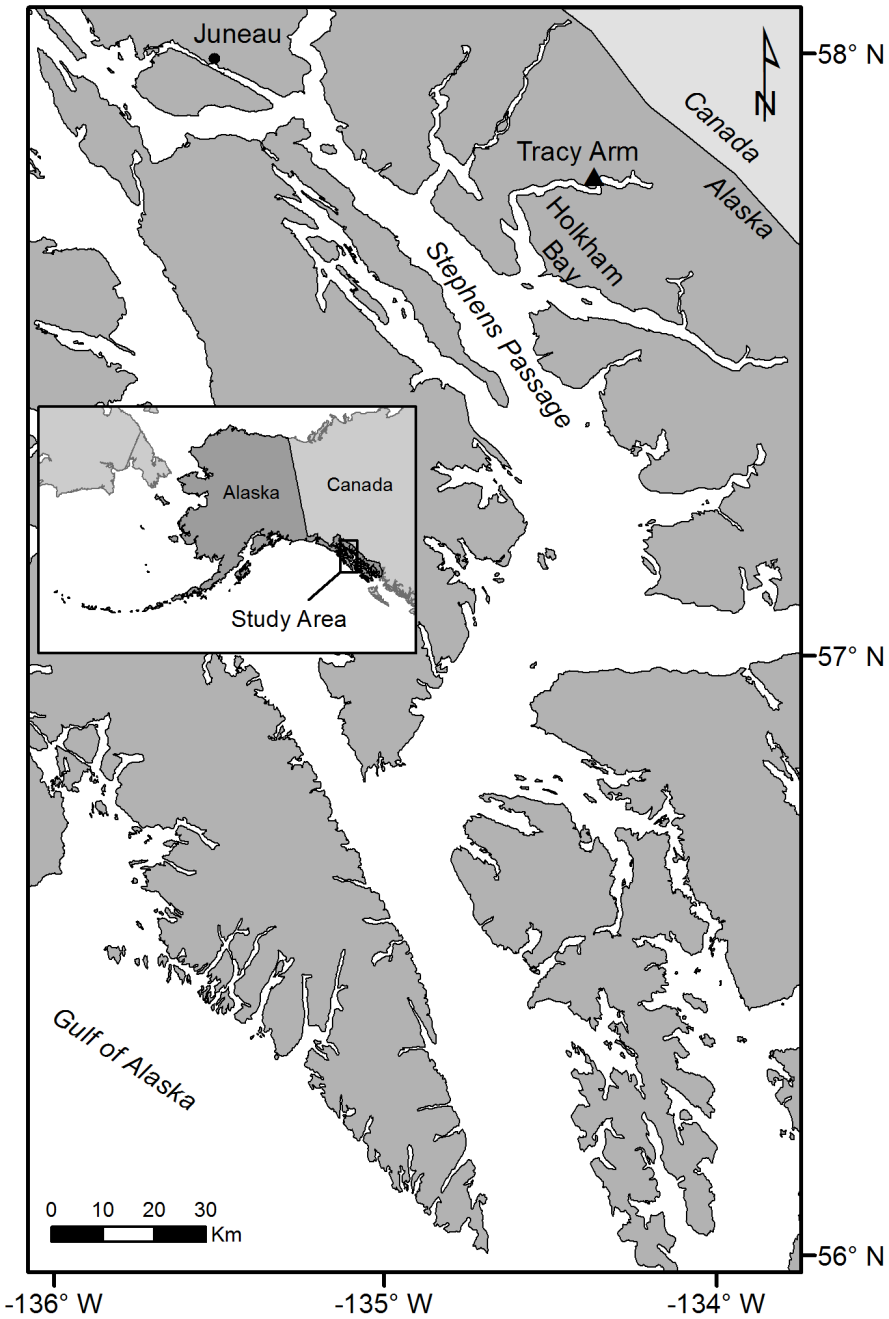
Study site in Southeastern Alaska. The study site in Tracy Arm, Holkham Bay, Southeast Alaska (indicated by the solid triangle symbol). The reproductive ecology of 38 *Primnoa pacifica* colonies were studied at the site between September 2010 and January 2012.

The study site is located in Tracy Arm, Holkham Bay, Southeast Alaska (Fig. 1). Tracy Arm is a 49-km long, deep (up to 378 m) glacial fjord that terminates in two tide-water glaciers. No permissions were required to work at this site. Tides in this region are semi-diurnal with a tidal range of more than 7 m. The study site consists of graywacke and granodiorite vertical walls ranging from high above sea level (the cliff directly above the site is ∼450 m and the full mountain peak ∼750 m) t o the fjord floor at a depth of almost 300 m. Red tree corals (a non-CITES species) were discovered thriving in several shallow water areas of the fjord in March 2006. The study site contains a relatively large thicket covering a horizontal distance of about 85 m and is located on the north side of the fjord approximately 36 km from the terminal moraine that forms a sill guarding the fjord entrance to Holkham Bay. The study site is approximately 13.4 and 15.4 km from the Sawyer and South Sawyer Glaciers, respectively. Previous observations from scuba and remotely operated vehicle operations indicate that the fjord contains several thickets, small patches and isolated red tree corals from depths of less than 10 m to at least 100 m.

### Sample Collection

Thirty-eight healthy (i.e. undamaged) colonies (42 to 162 cm total length along the longest axis) were haphazardly selected for tagging between 8th and 10th September 2010. Colonies were tagged above the holdfast with numbered, plastic cinch-type tags by scuba divers. The depth range for all tagged colonies was 9.8 to 17.1 m mean lower low water (all depths reported hereafter are referenced to mean lower low water). Reference images were collected of each colony with a meter stick scale, and Image J (National Institutes of Health) was used to calculate flat-plane area of colonies.

Samples were collected from all 38 tagged-colonies during six time periods over an 18-month span: September 2010, December 2010, March 2011, June 2011, and January 2012. In September 2011, extremely poor visibility limited us to collected samples from only 10 colonies (9 females and 1 male). The poor water visibility was due to heavy sediment loading and corresponded with warm air temperatures and the onset of the rainy season in the region—both factors increasing stream run-off and glacial melt.

Pieces approximately 4–7 cm in length (containing ∼30 polyps) were carefully sampled from a distal branch of each tagged colony. Samples were isolated in individual jars *in situ* and returned to the surface where they were kept in containers of cold seawater until onboard processing. The number of polyps per 1 cm was counted for each sample collected, averaging over a 5 cm span of skeleton.

Ten of the 38 colonies were randomly selected following the initial sampling (September 2010) for the simulated physical damage treatment group. Physical damage consisted of tearing a large branch(s) from each colony that comprised 21–40% of the total colony volume. This degree of damage is consistent with that observed *in situ* in the Gulf of Alaska (Stone et al., in preparation). Removed tissue was retained, photographed on a scale board on deck, and the dry weight was obtained in the laboratory. Image J (NIH) was used to calculate the flat-plane area of the removed branches.

### Environmental Monitoring

A Star-Oddi DST-CTD (mention of trade names or commercial companies is for identification purposes only and does not imply endorsement by the National Marine Fisheries Service, NOAA) compact microprocessor-controlled temperature, depth and salinity recorder was attached to one of the tagged colonies during the first field season (Sept. 2010) and was retrieved during the final field season (Jan. 2012). The recorder was programmed to record measurements every 6 hours and the depth of deployment was 14.9 m.

During each sampling period a Seabird Electronics Seacat Profiler was used to collect depth, temperature, salinity, and density profiles of the water column to a depth of 50–120 m. Profiles were collected directly over the primary study site and one in the center of the fjord.

A Sontek Argonaut-MD Doppler current meter equipped with an internal compass was moored on an untagged colony (depth = 11 m) near the in-fjord end of the study site. The meter was programmed to record average current velocity and direction every 10 minutes. The instrument was deployed between 10 March 2011 and 3 January 2012.

### Live Observations

Two to 5 cm pieces of each tagged coral colony were dissected live aboard the research vessel during the March 2011 and September 2011 cruises. Oocytes and spermatocysts were extracted from polyps using a Leica S8APO stereo microscope, observed and photographed (Motic 10 camera attachment, Motic Optical Inc.) for 24 to 48 hrs.

### Histological Processing

Eight centimeter pieces of all 38 tagged corals were preserved in 10% neutral-buffered formalin for 24–48 hrs and then washed and transferred to 70% ethanol for long-term storage. From these pieces, two sets of three to five polyps were dissected from each specimen for histological analysis.

Polyps were decalcified using RDO Rapid Decalcifier (Apex Engineering Products Corporation) until all spicules were dissolved. These samples were then dehydrated to 100% ethanol, using a graded ethanol series (10–20% increments), and cleared using toluene. Samples were then infiltrated in molten paraffin wax for 24 hrs and embedded using standard histological molds. Five micron sections were cut for both fecundity (serially every 90 microns) and for oocyte size analysis (five centrally located slides) on a Leica RM2235 rotary microtome. Sections were stained with Masson's Trichrome or Haemotoxylin - Eosin (H&E) using standard procedures, and permanently coverslipped. Each slide was examined under an Olympus CX21 microscope, with Moticam 5 camera (Motic Optical) attachment used to grab images. Images were analyzed using Image J (NIH).

### Analysis

Male to female ratio of the study population was calculated based on the 38 tagged colonies. For each female colony, one hundred random oocytes were measured per collection (measuring only oocytes with nucleus present to avoid measuring the same oocyte twice), and seasonal oocyte-size frequency graphs were constructed using feret diameters (the calculated diameter as if the oocytes were a perfect circle). Mean oocyte-size for each colony was also recorded, and three polyps per female had all previtellogenic and vitellogenic oocytes counted for fecundity estimations. For male colonies one hundred random spermatocysts were staged, and seasonal bar charts constructed with percentage of each stage per colony.

To examine for differences between simulated damage colonies and non-damaged colonies, Mann-Whitney U tests were conducted to assess differences in average oocyte size; average fecundity and average spermatocyst stages. For comparisons, non-damaged colonies of similar size to damaged colonies were used. Non- parametric Friedman tests (SPSS 2012) were conducted on mean oocyte sizes and fecundity in females and between average spermatocyst stages in males, to test for synchronicity between individuals within and between months. Total monthly mean oocyte size was then calculated, and a Pearson product moment correlation (SPSS 2012) was used to examine oocyte size correlations and percentage of late-stage spermatocysts with mean monthly temperature from the recorder.

## Results

### The Fjord Environment

**Figure 2:**
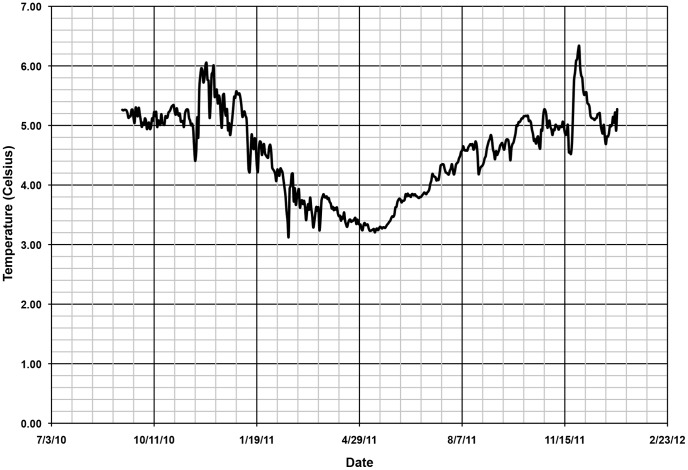
Water temperature records from study site. Average daily water temperature measured at a depth of 15

The salinity cell on the Star-Oddi recorder failed, possibly due to fouling, during the first month of deployment, therefore only temperature data were available for the full length of the study (Fig. 2.). Average daily temperatures at 15 m depth ranged from 3.14 to 6.33°C over the course of this study. The coldest average daily temperature was recorded in February 2011 and the warmest in late November 2011. The general trend was 1) cooling in early December and 2) warming beginning in early May. CTD casts taken during research cruises confirmed these temperatures at 15 m depth. Salinity from CTD casts showed a high of 31.41 in March 2011 and a low of 26.86 in September 2011. Dissolved oxygen readings were highest in the cooler months (7.66 mg/l in March 2011) and lowest in the warmer months (4.07 mg/l in December 2010). Water currents were generally bi-directional with the strongest currents (up to 12 cm sec^−1^) out-fjord with a weaker reciprocal current in-fjord.

### Tagged Colonies

Of the 38 colonies collected, 20 were females (45–162 cm axial length), 17 were males (42–151 cm axial length) and one was non-reproductive (42 cm axial length). Due to the absence of true gonads in cnidarians, it is impossible to classify the non-reproductive colony as male or female. The sex ratio of this population was therefore 1.17∶1 females∶males. The average number of polyps per centimeter (axial length) was calculated as 7.96 (SE ±1.22). The largest colony in our study had a photographed surface area of around 3500 cm^2^, and the smallest colony around 800 cm^2^.

### Internal Morphology

**Figure 3:**
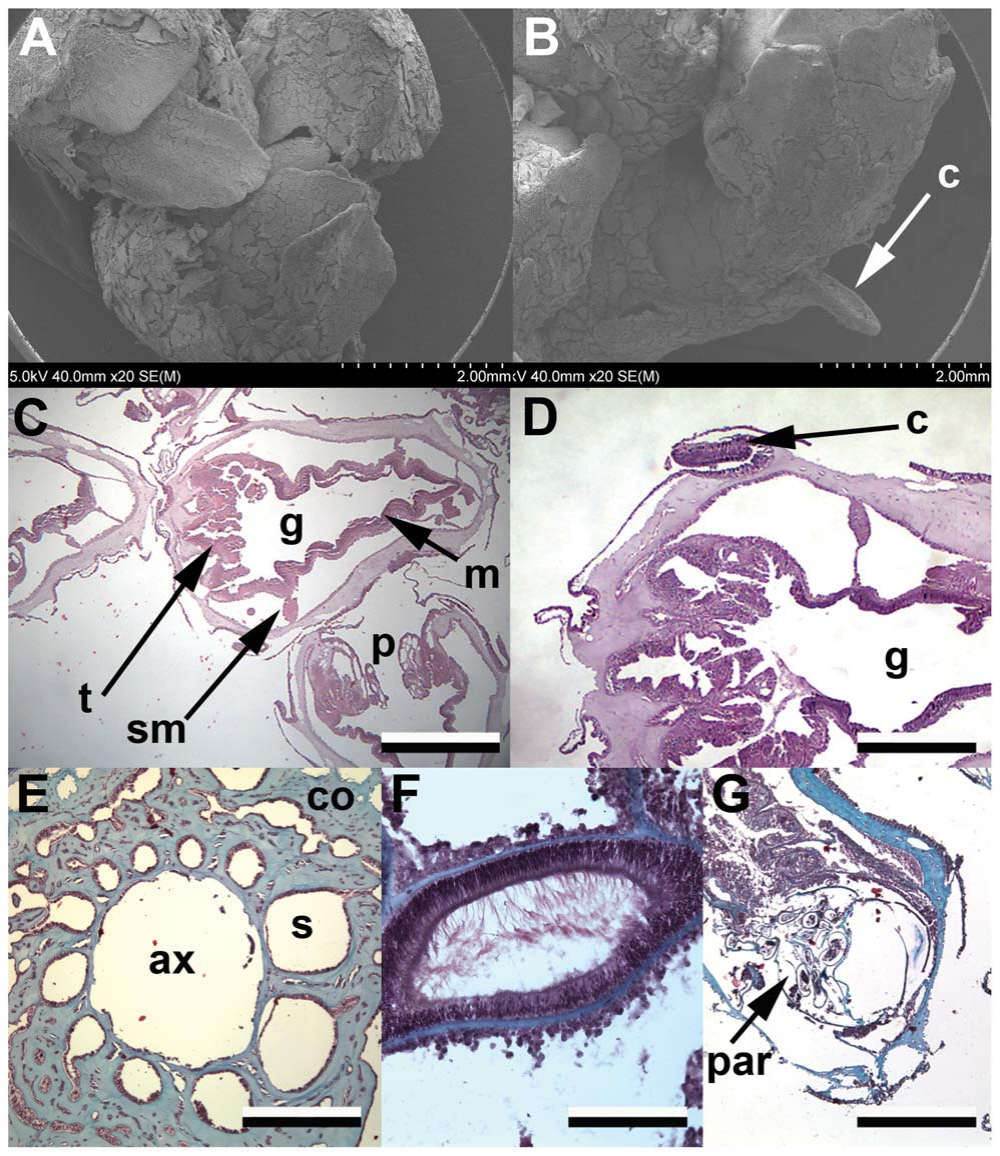
External and internal anatomy of red tree coral. *Primnoa pacifica* morphology. a) SEM image of three axial polyps; b) SEM image of single axial polyp with horny collar (c) shown; c) Histological image of whole polyp, scale 1000 microns; d) Histological section showing muscular attachment for horny collar, scale 500 microns; e) Histological section showing axis of the colony, scale 500 microns; f) Histological section showing siphonoglyph, scale 200 microns; g) Histological section showing parasite at base of polyp, scale 1000 microns. (c, horny collar; t, tentacles; g, gastrovascular cavity; sm, septal muscle; p, polyp; m, muscle; ax, axis; co, coenenchyme; s, solenia; par, parasite).

Results from this study confirmed that *Primnoa pacifica* is monomorphic, with asexual reproduction occurring by extratentacular budding along the central gorgonin axis, and sexual reproduction occurring within the autozoids. This morphology is similar to that found in most other gorgonians [25]. Polyps were non-retractile and were heavily armoured in sclerites (Fig. 3a,b). Externally located (Fig. 3b,d), a horny collar structure was found on either side of the polyp (2 per polyp). The function of this structure is unknown but it was formed through a thick layer of epidermal cells and appeared to be protected by a large outer sclerite (Fig. 3b). The internal layers of this structure were thickened, with one side containing a brush border, and the other producing mucus.

The study confirmed that the internal anatomy of *P. pacifica* was common to that seen in other gorgonians (Fig. 3c–f). Polyps had a short pharynx and short thick tentacles, with a large retractor septal muscle, leading to the gastrovascular cavity. Lining the gastrovascular cavity were eight mesenteries, where the gametes form, and a prominent siphonoglyph, with long cilla (Fig. 3f). The coenenchyme that connects individual polyps together (and to the axis) was thick, and filled with solenia tubes (Fig. 3e).

Unidentified crustacean parasites were occasionally observed at the base of polyps (Fig. 3g). Polyps that contained parasites did not contain gametes (or contained few gametes) and so were not utilized for reproductive morphology, periodicity and fecundity estimates. These polyps were typically situated next to reproductively active polyps without parasites.

### Reproductive Morphology

**Figure 4:**
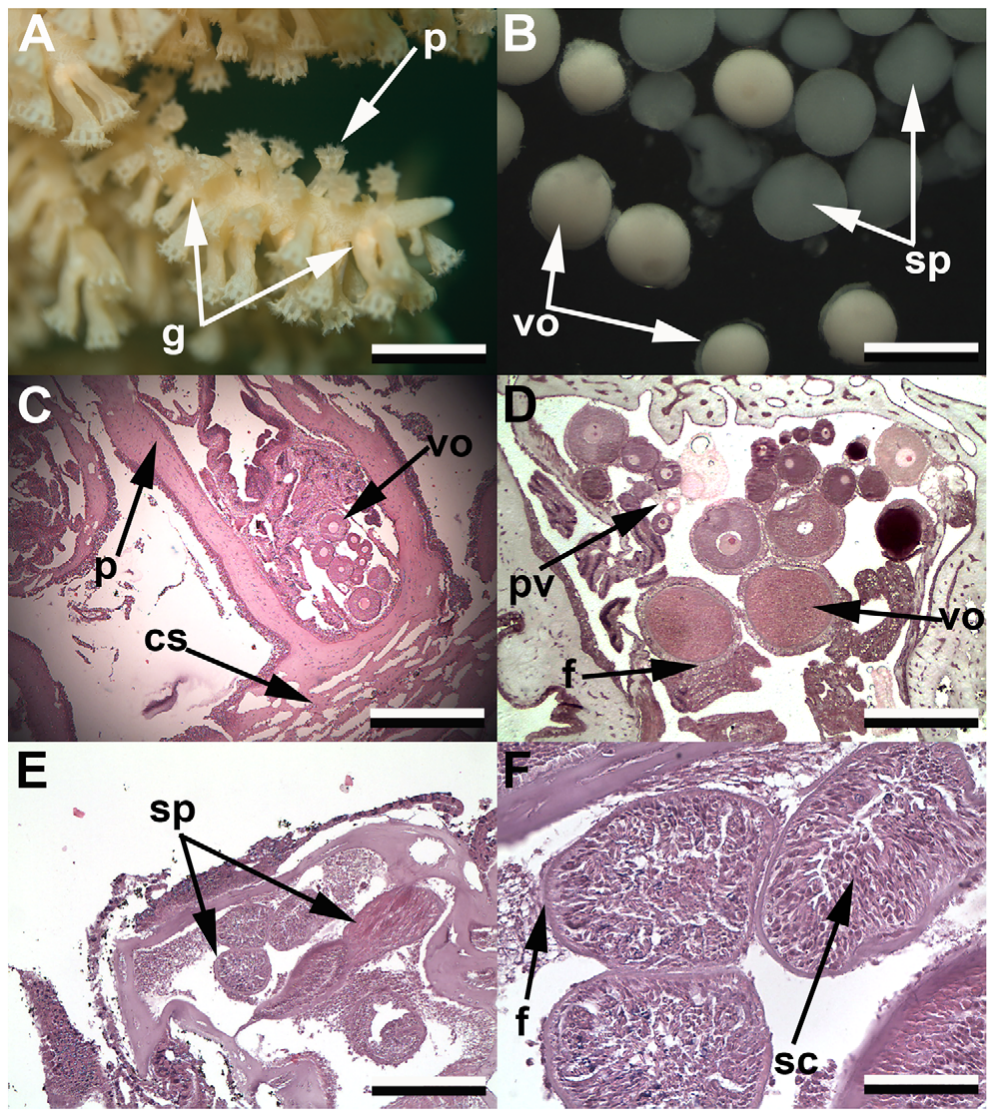
Reproductive anatomy of red tree coral. *Primnoa pacifica* reproductive anatomy. a) *In situ* photograph of open polyps showing gametes at base (g), scale ∼3 cm; b) Extracted oocytes (vo) and spermatocysts (sp), scale 2 cm; c) Histological section showing female polyp, scale 1000 microns; d) Histological section of female polyp, scale 500 microns; e) Histological section of male polyp, scale 200 microns; f) Histological section of spermatocysts, scale 100 microns. (g, gametes; p, polyp; vo, vitellogenic oocytes; sp, spermatocysts; cs, coenchyme; pv, previtellogenic oocytes; f, follicle cells; sp, spermatocysts; sc, spermatocytes).

*Primnoa pacifica* is a gonochoric coral, with colonies being either wholly male or female. Hermaphrodites, either simultaneous or sequential, were not found during this study. Gametes were located at the base of polyps in bundles forming off the eight mesenteries (Fig. 4a,c), with each gamete being surrounded by follicle cells that thickened as the gametes developed (Fig. 4d,f). Once fully mature, gametes were found detached from mesenteries, high in the gastrodermal cavity, presumably for release. Multiple stages of both oocytes and spermatocysts were often present within single polyps.

Through live dissection, both ripe oocytes and spermatocysts (spermaries) were extracted and observed through the stereomicroscope (Fig. 4b). Vitellogenic oocytes were light peach in color and an indentation of the germinal vesicle was observed on the periphery of each oocyte (Fig. 4b). Spermatocysts were an opaque white color and tended to be slightly larger than ripe oocytes (Fig. 4b). These cysts remained intact for approximately 24 hrs when incubated in seawater collected at depth and held at ambient water temperatures, then began to disintegrate, releasing sperm into the water. Fertilization of oocytes was not observed during study.

Oocyte sizes were measured for histological analysis. Previtellogenic oocytes measured ranged from 20–220 µm feret diameter before undergoing vitellogenesis. Vitellogenic oocytes ranged from 220 µm to 802 µm. The nucleus started centrally in the previtellogenic oocytes, and moved peripherally as the oocytes ripen, with trophonema becoming apparent in larger oocytes. All oocytes were surrounded in follicle cells, with vitellogenic oocytes having the thickest surrounding layer. No larvae were identified in the histological sections and none were observed anytime during this study, though in several dissections unfertilized oocytes were observed adhered to the outside of female polyps in the medial area facing the axis.

Spermatocysts measured through histology ranged from 500–1000 µm in feret diameter. Spermatocysts were surrounded by a membrane, creating a sac off the mesentery where sperm form (Fig. 4e). This sac had a thin layer of follicle cells surrounding them throughout their life cycle. Four stages of spermatocysts were identified – Stage I showed a loose aggregation of tailless spermatocytes (and presumably spermatogonium); Stage II showed more organization of spermatocytes, with a loose lumen being formed; Stage III consisted of organized spermatocytes and spermatids around a distinct, but empty lumen; and Stage IV spermatocysts had lumen packed with sperm tails and were often found detached from the mesentery tissue within the gastrovascular cavity.

### Simulated Damage

Histological analysis showed that 6 of 10 colonies with simulated damage were male and 4 were female (with one female and one male being juvenile, with just a few early stage gametes present). Image analysis (Image J, NIH) showed that between 21–40% of the tissue mass was removed from each of these colonies. None of the damaged colonies showed any indications that reproduction was affected. In comparison to the undamaged colonies, neither fecundity (U = 6, *p*≤0.05) or oocyte size (U = 2, *p*≤0.05) was affected in females; nor was spermatocyst development in males (U = 3, *p*≤0.05). As there was no significant difference between simulated damage colonies and non-damaged colonies, the results from these colonies were pooled with the non-damaged for the remaining analysis.

### Fecundity

**Figure 5:**
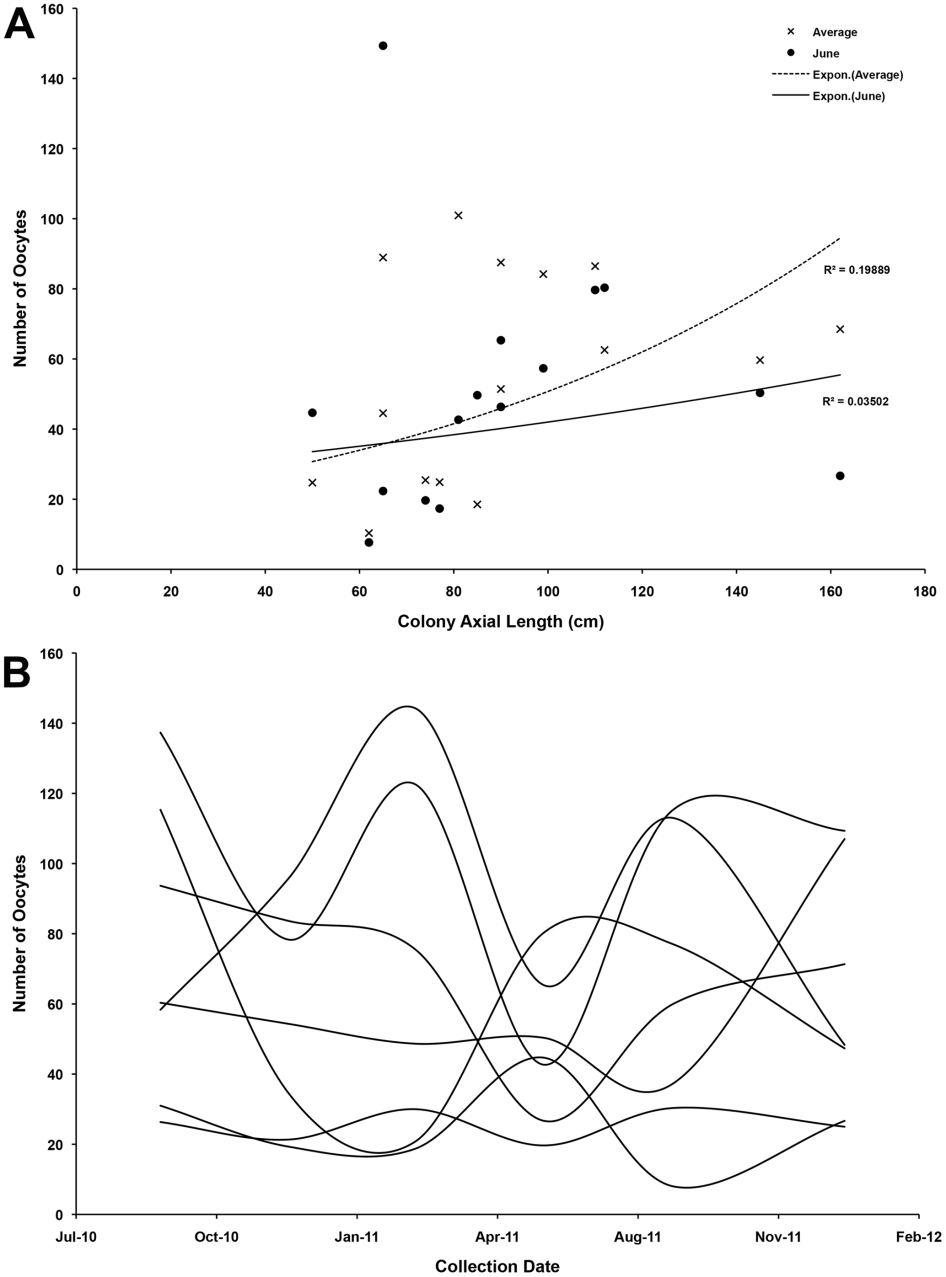
Fecundity measures for red tree coral. A) Size specific fecundity for *Primnoa pacifica* colonies. Mean total fecundity (dark) averaged over the six time points, and June mean fecundity (open) used. Neither measurements show a positive correlation with axial length; B) Mean total fecundity over months analyzed for seven individuals showing varied patterns per colony.

Average polyp fecundity for undamaged colonies was 86 (SE±23) total oocytes, with 69 (SE±14) previtellogenic and 17 (SE±12) vitellogenic. There was a slight trend of increased fecundity with colony size but the relationship was not significant (Fig. 5a, R = 0.24377 & R = 0.025923). The smallest reproductively mature colony was a male at 42 cm length. The photographed area of each coral colony was calculated using ImageJ (as above), thus an approximate number of polyps was calculated per colony. The largest colony in our study had an approximate potential (i.e. ripe, vitellogenic oocytes) fecundity of 475,000 oocytes, and the smallest female mature colony a potential fecundity of 110,000 oocytes.

### Periodicity

**Figure 6:**
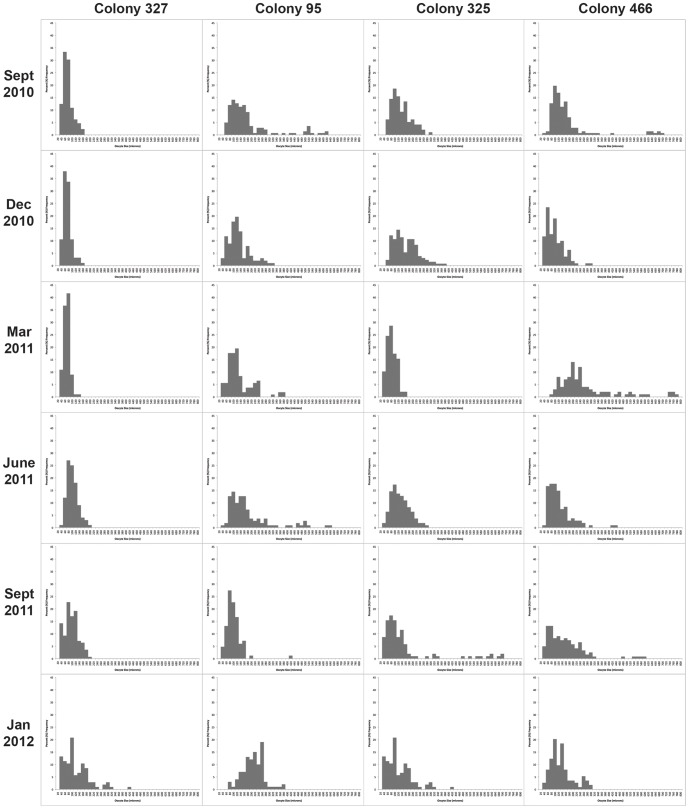
Seasonal oocyte size frequency graphs. Examples of seasonal oocyte size frequency graphs for four individual colonies collected in this study.

**Figure 7:**
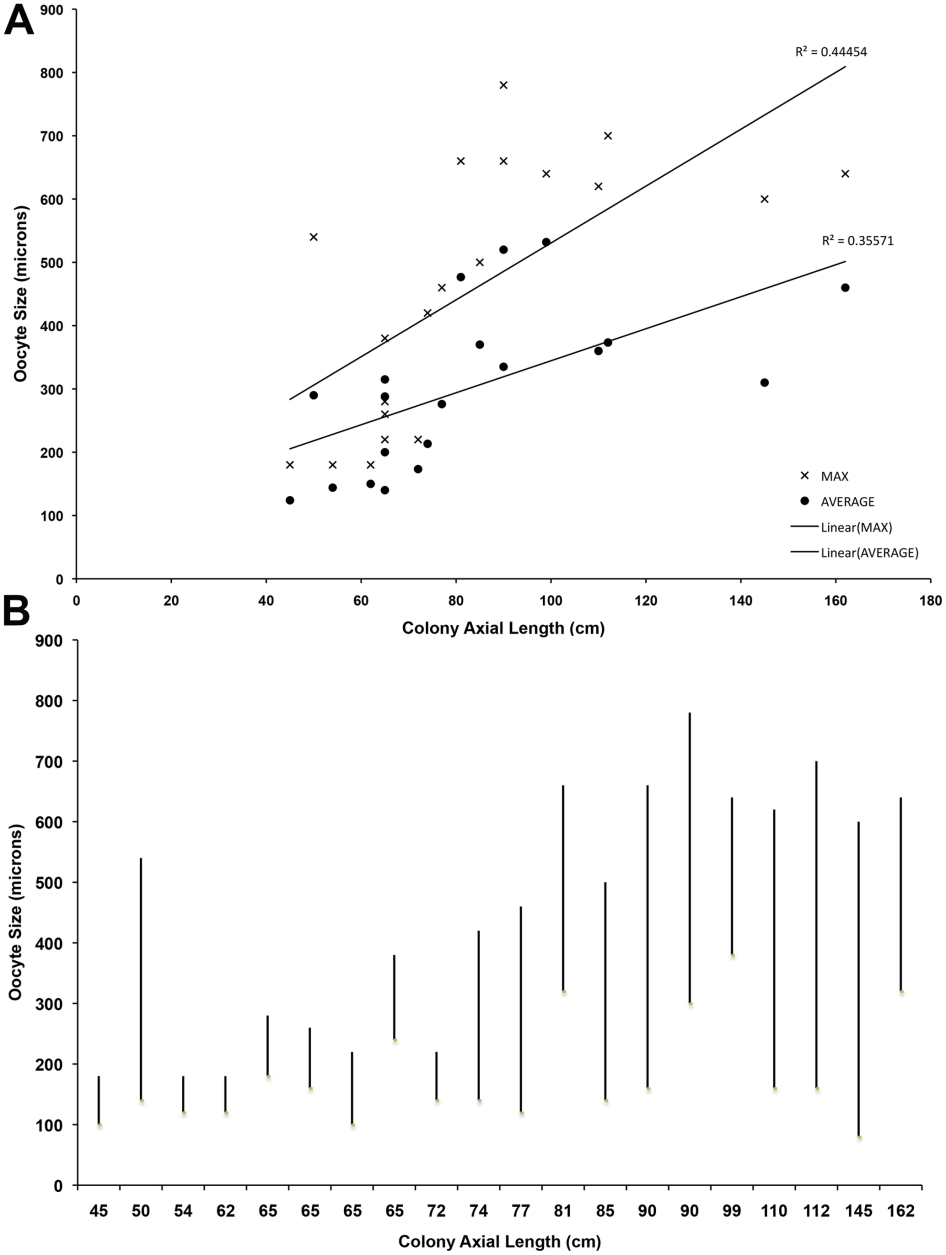
Oocyte size analysis. A) Range of oocyte sizes plotted against length of colony. Logarithmic trendlines are shown; B) Maximum and minimum oocyte sizes plotted against colony length.

Oocyte size frequency patterns (Fig. 6) were highly variable across the population. Size frequency plots suggest that spawning is largely asynchronous between individuals in this population. A Friedman test was performed between colonies to test for asyncronicity, with a Bonferroni correction for multiple comparisons. Mean oocyte sizes were significantly different over the months sampled (X^2^ = 12.929, *p* = 0.044), although no pairwise comparison was significant, potentially due to the low sample size. Average and maximum oocyte sizes were plotted against total colony length (Fig. 7a), and slight correlations were seen in both categories (R^2^ = 0.51 for maximum; R^2^ = 0.43 for average). Fecundity of vitellogenic oocytes appeared to be on more than a yearly cycle (Fig. 5b), though as with oocyte sizes, there was great variation between individuals. Plotting the minimum and maximum oocyte sizes versus colony length (Fig. 7b) showed a clear trend for smaller colonies to produce smaller oocytes up until a length of around 80 cm was achieved.

**Figure 8:**
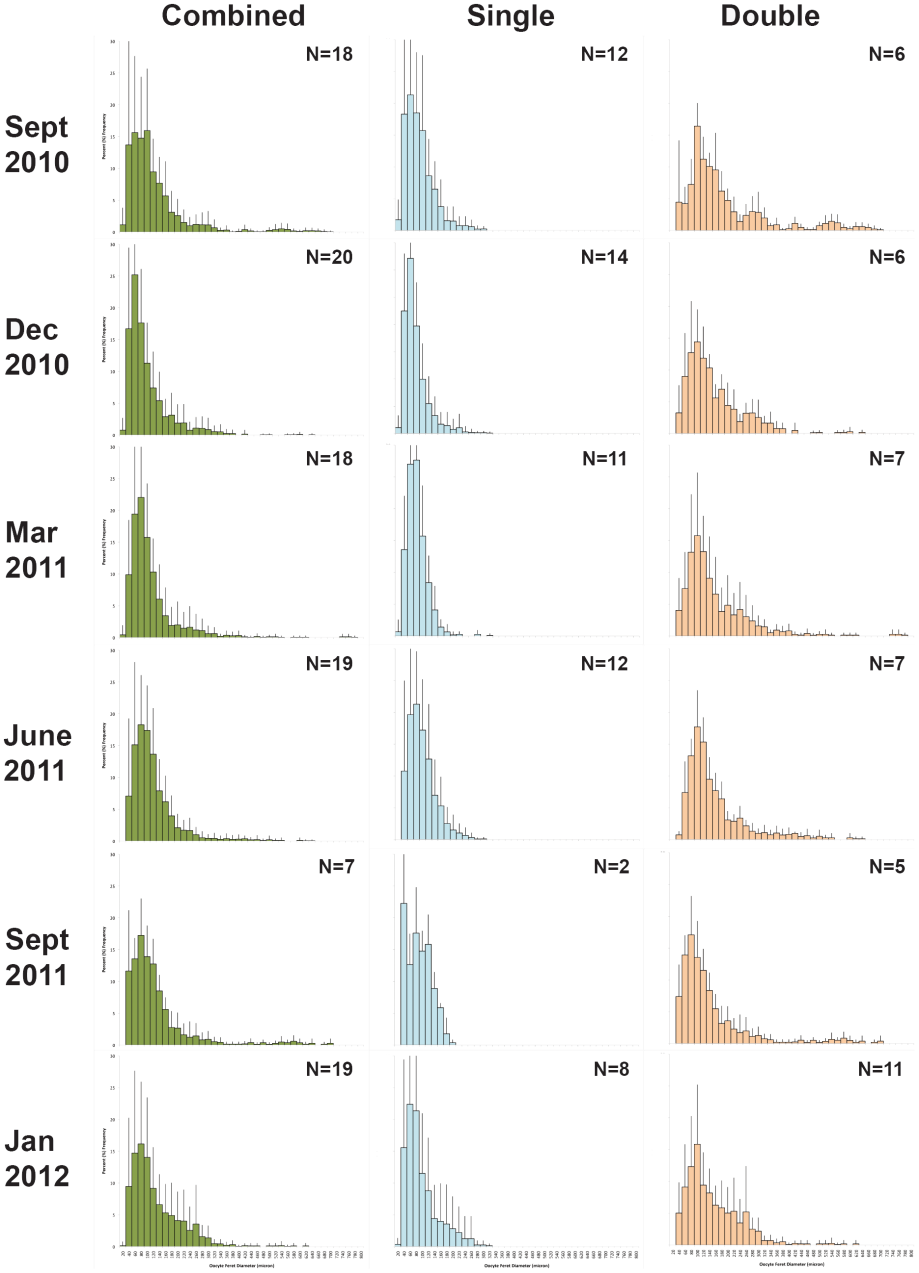
Seasonal oocyte size frequency graphs. Oocyte size frequency data – Combined - all females with oocytes present averaged; Single – all females with only previtellogenic oocytes; Double – all females with both previtellogenic and vitellogenic oocytes. N = number of colonies combined.

All individual oocyte size frequencies were assessed visually and, after creating combined monthly graphs, were split into individuals of similar patterns (Fig. 8). In each month examined there was a percentage of mature females with vitellogenic oocytes present (‘double cohorts’) and a percentage with only previtellogenic oocytes present (‘single cohorts’). The percentage of individuals showing single and double cohort traits differed in each month, with a higher percentage of mature females in January 2012, and the lowest percentage of mature females in September 2011 and 2010. This suggests a larger spawning event early in the year than in the fall months.

**Figure 9:**
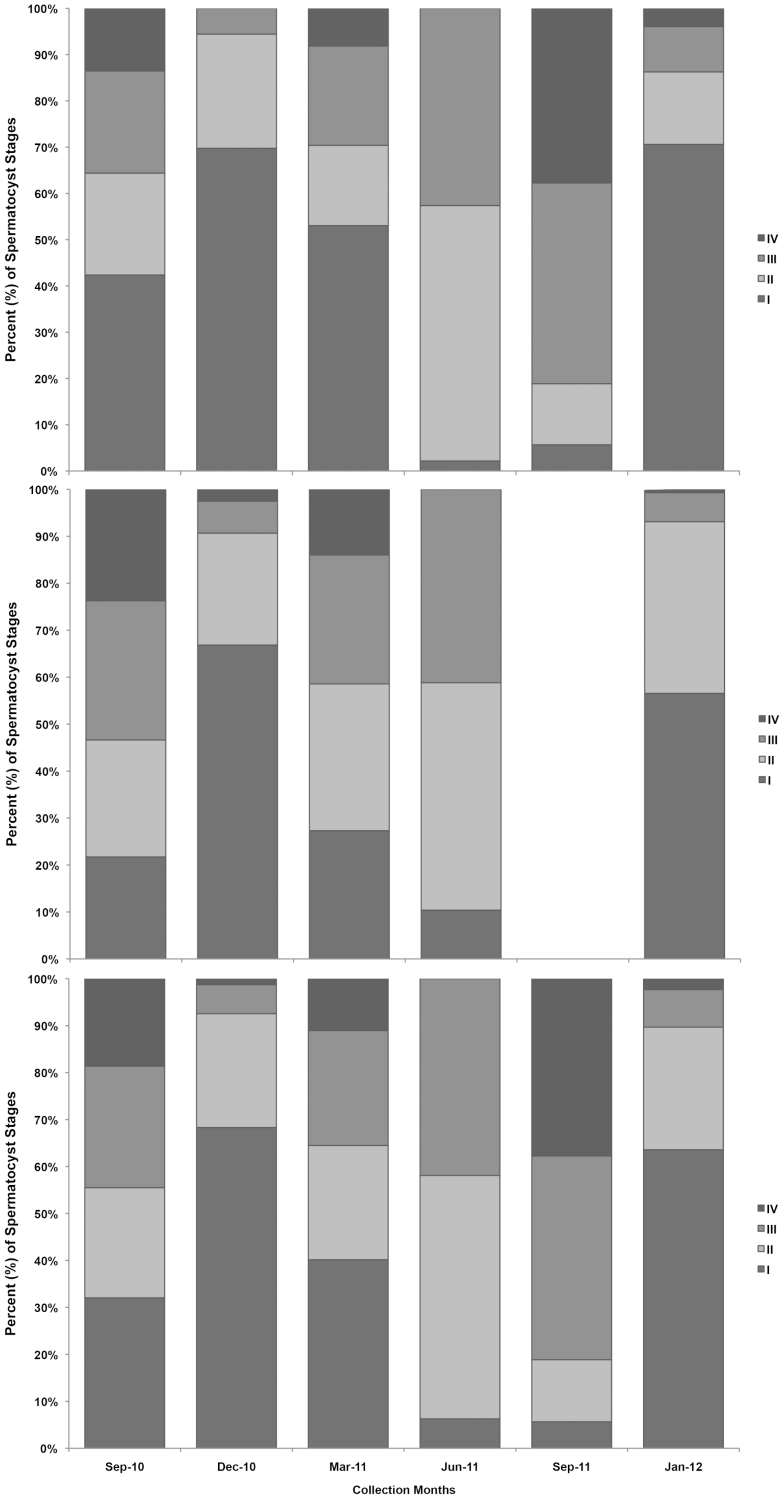
Seasonal male analysis. Stacked bar graph showing stages of spermatogenesis over sampling periods analyzed. a) Non damaged colonies; b) simulated damage colonies; c) combined means.

One hundred random spermatocysts per colony, per sampling period were staged using the same scale as above (Fig. 9a–c). Figure 9 shows the percentage of spermatocysts at each stage in undamaged colonies (a); simulated damage colonies (b); and all male colonies (c). In both years, September appeared to have the highest percentage of late stage spermatocysts, while June and September 2011 had the lowest percentages of early-stage spermatocysts. In all colonies measured, June 2011 had no late-stage spermatocysts present in polyps. For males, September 2011 was removed from statistics because no ‘non-damaged’ males were collected that month.

There was also no correlation between mean oocyte sizes (r = 0.205, *P* = 0.05), mean fecundity (r = 0.077, *P* = 0.05), or the percentage of late-stage spermatocysts (r = −0.048, *P* = 0.05) with water temperature.

## Discussion

The goals of this study were to describe the mode and periodicity of reproduction for one of the most abundant and widespread deep-sea corals in the North Pacific Ocean. We studied a population of deepwater emerged *Primnoa pacifica* and in addition assessed whether reproduction in this species is affected by sub-lethal trauma so as to gauge its ability to sustain populations threatened by disturbance from fishing activities. We collected two hundred and 28 samples from 38 tagged colonies over six sequential seasons and examined more than 10,000 histological sections. This dataset represents one of the more extensive reproductive studies for any cold-water coral species in the world, and provides higher resolution (i.e. same colonies resampled over time) data than previously collected to our knowledge.

Mass spawning events are a common trait amongst broadcast spawning corals, providing for greater genetic diversity, protection from over-predation and enhancing fertilization success [26], [27], [28]. In zooxanthellate and azooxanthellate octocorals mass spawning events have been tied to temperature, lunar cycles and food availability [29], [8], [30], [11], [31]. This population of *P. pacifica* does not appear to fit with either a mass spawning event, or quasi-continuous spawning events documented in other octocorals [32], [33], [11], [34] or scleractinians (see review in [28]). Intra-colony variation was not observed in this study (i.e. all polyps appeared to have similar gamete stages and fecundity); however, there was great asynchronicity of inter-colony gametogenesis and apparent spawning events. Some females appeared to mature oocytes twice a year, whereas others only matured oocytes once over the 16 months sampled or appeared to have a continuous supply of mature oocytes (Fig. 6 and Fig. S2). Duration of gametogenesis tends to be ‘long’ in many octocoral species, primarily because larger, energetically expensive, oocytes tend to be the norm [35]–[37]. *Primnoa resedaeformis* from the North Atlantic appear to have all stages of oogenesis in each polyp, suggesting overlapping cohorts or quasi-continuous oogenesis in this closely related species [38]. No males were observed in that study but Mercier et al. [38] also found that oocyte sizes were similar (though larger, at 1000 microns) as was fecundity and reproductive mode (i.e. no larvae were observed), showing potential conservation of traits amongst the genus [34].

Male colonies did appear to show some periodicity to sperm maturation (Fig. 9), with potentially three spawning periods observed during this study – September 2010 through December 2010, March 2011 through June 2011, and again between September 2011 and January 2012. At the beginning of the study, in September 2010, male colonies had all four stages of spermatocysts present in nearly equal percentages. By December 2010 many of the late-stage spermatocysts had disappeared, presumably through spawning (though this was not observed), and more early-stage spermatocysts were being developed. In March 2011 many of these early stages had developed into Stage IV spermatocysts, which were then spawned off by the June 2011 collection. Then in September 2011 (with the caveat this was a low collection season) earlier stage spermatocysts appear to quickly ripen for spawning through January 2012. Although there are three spawning cycles observed in this study (two within a single year), the percentages of early-stage spermatocysts available for development appeared to have their own pattern. This cycle appeared to take just over a year to complete – early development occurred through September 2010, and large numbers were not regenerated again until somewhere between September 2011 and January 2012.

Although spawning periods for males and females might occur throughout the year, corals may only be able to produce germ cells for spermatogenesis on a yearly cycle, and may only be able to mature oocytes through vitellogenesis sporadically, maybe when conditions are suitable for spawning. The trophic ecology of this species is unknown, but there are ties between food availability, lipid synthesis and reproduction in cold-water octocoral species [39], [40]. This temperate ecosystem is strongly influenced by seasonal pulses of plankton but given the steep fjord wall environment the corals inhabit, they are not able to rely on the predictable ‘food bank’ afforded to other temperate and polar communities inhabiting horizontal seafloor to continue reproduction year-round [41], [42]. The study area is subjected by a strong predictable spring phytoplankton bloom each year, with minor subsequent blooms during the summer months [43]. We observed no obvious coupling of reproductive events for corals in this study and the timing (April) of the spring phytoplankton bloom and therefore conclude that the timing of reproduction in this species is controlled by other factors, possibly a complex suite of environmental factors. Though different octocoral species exhibit different strategies for energy storage [40], we can hypothesize that this population of *P. pacifica*, with its ability to reproduce with marked inter-colony asynchronicity, permanent availability of previtellogenic oocytes for maturation and highly seasonal food availability, would be efficient at both storing and potentially quickly utilizing food resources for reproductive purposes. Studies into energy investment and storage in this species would be highly valuable to understanding the wider implications of their high reproductive outputs, and their ability to sustain populations in both deep and shallow ecosystems.

These data provide important and timely insights regarding the capability of some of the region's dominant benthic epifauna to recover from disturbance and to recolonize areas previously disturbed by human activities. The recovery rate of disturbed benthic ecosystems in the Gulf of Alaska is of keen interest to fisheries managers given recent actions in the region to preserve areas of known coral abundance [44], including *P. pacifica* thickets [4]. Large *Primnoa* colonies (>2 m height) are rare in the Gulf of Alaska and are much more susceptible to damage from fishing gear. In contrast to what has been shown in several other studies (e.g. [45], [46], [47], [12], [13]), our comparison of undamaged and damaged colonies did not indicate any adverse effects to reproduction from sub-lethal physical trauma. However, sub-lethal trauma, like that simulated in this experiment, reduces the overall fecundity of colonies in direct relation to the amount of the colony that is removed.

Our results also indicate that colonies smaller than 42 cm in axial length are non-reproductive and that female colonies smaller than ∼80 cm produce smaller oocytes than larger colonies. Oocytes are energetically expensive to produce, particularly the larger (∼300–1000 µm) yolky eggs that appear to be characteristic of octocorals [36]. Oocyte size can influence fertilization success in marine invertebrates, with sperm availability influencing optimal oocyte sizes for individual species [48]. In this population, smaller colonies produced smaller oocytes than larger colonies, creating a dichotomy of oocytes sizes within a single population. Though there have been some reports of different populations having different oocyte sizes (creating genetic subdivision, [49]); these differences appear rare [48] and we could find no reports of different oocyte sizes within a single population. In urchins, some species of *Strongylocentrotus* with smaller oocytes require a higher concentration of sperm to fertilize at equal rates to species with larger oocyte sizes, though species from a different genus show no differences (reviewed in [48]). For this population from Tracy Arm fjord, if larger colonies produce the ‘optimal’ size of oocyte for the relative sperm availability and adaptation, then oocytes produced by smaller colonies could have less fertilization success than those of larger size. This could have an overall effect on total population fecundity should colonies be broken to smaller size classes through fisheries damage.

Given the long duration of gamete development and seemingly long recruitment times (i.e. no new recruits were observed in this study area, pers. obs), removal of polyps able to produce germ cells and mature gametes would be directly detrimental to population sustainability. Removal of entire colonies is lethal and effectively removes the entire reproductive output for those colonies and results from this study indicate that recruitment events are likely to be highly sporadic in this species. In addition polyp level fecundity has been found to be related to overall population density in other cnidarians including gorgonians and pennatulids [36]. Discrete areas of with abundant, large *P. pacifica* colonies would be good candidates for reproductive refugia such as the Habitat Areas of Particular Concern (HAPCs) that were designated by the North Pacific Fishery Management Council in 2007 [3], [4].

Primnoidae are the most abundant corals in Alaskan waters [50]. These findings might be applicable to the other 18 species in this family found in Alaskan waters. Kahng et. al. [34] reported that sexuality and reproductive mode tend to be conserved in families amongst octocorals, with gonochorism and internal brooding being dominant traits in the Primnoidae. The results of this study confirmed that *P. pacifica* is indeed a gonochoric species but it not an internal brooder. We found on several females polyps where unfertilized oocytes were “stuck” to the epidermis of the polyps in a central area. We also question the purpose of the “collar” structure. This thickened epiderm is potentially lined with a brush border, filled with mucus and protected by sclerites, making it an ideal protected environment for external brooding. Decalcification for histology would loosen this areas hold on gametes or larvae, potentially losing firm identification of this structures function. Only further studies through electron microscopy might elucidate the function of this structure.

## Conclusions

This study focused on an accessible shallow-water population of *P. pacifica*, yet this species is more commonly found in deep-water (125 to 800 m) in the Gulf of Alaska. The purpose of this study was to provide baseline data from which to compare deep-sea samples in the future, as well as provide information to evaluate the potential effectiveness of management measures for this species such as designation of Habitat Areas of Particular Concern [3], [4]. In this shallow water situation, *P. pacifica* shows a spawning reproductive mode, with oocyte and spermatocyst development generally taking over a year for a full cycle. Though spermatocyst development appears synchronous, oocyte development is highly asynchronous with multiple spawning events occurring during a single year (2–3 events observed during this study). This strategy would allow for enhanced genetic diversity, and, for a population living at the extreme edge of the species bathymetric range, within generation bet-hedging on when in the year optimum larval development, survival and recruitment would occur [51]. In the deep ocean, where seasonal cues can be more discrete and limited, it has been found in other species that though sexuality and reproductive mode is constrained, fecundity can be reduced [52], [38], [53]. The results from this study will provide for a comparison with samples presently being collected from deep-water so that we can address questions regarding whether depth-related ecological effects occur in this species, and aid in forming management plans for those specific populations.

## Supporting Information

Figure S1 **Graph showing colony lengths against depth of colony.** Graph shows colonies selected covered a wide variety of sizes between our defined depth range; male and female colonies were spread out with respect to depth; and that colonies selected for simulated damage were selected haphazardly around the sample site. There is a non-significant trend towards larger colonies being deeper at this site (R^2^ = 0.434).
(TIF)

Figure S2 **Graphs of individual oocyte size frequency data.** Each graph is for a single individual with each month of data plotted.
(TIF)
