# Supplementary figures and images for: Sexual Reproduction and Seasonality of the Alaskan Red Tree Coral, Primnoa pacifica
Source: PLoS One. 2014 Apr 25;9(4):e90893. doi: 10.1371/journal.pone.0090893 (PMC4000209; doi:10.1371/journal.pone.0090893)

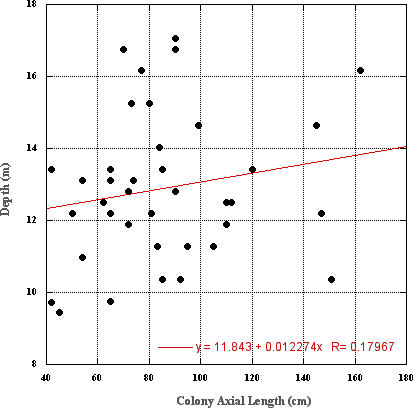

Supplement: Figure S1 — Graph showing colony lengths against depth of colony. Graph shows colonies selected covered a wide variety of sizes between our defined depth range; male and female colonies were spread out with respect to depth; and that colonies selected for simulated damage were selected haphazardly around the sample site. There is a non-significant trend towards larger colonies being deeper at this site (R2 = 0.434). (TIF) [file pone.0090893.s001.tif]

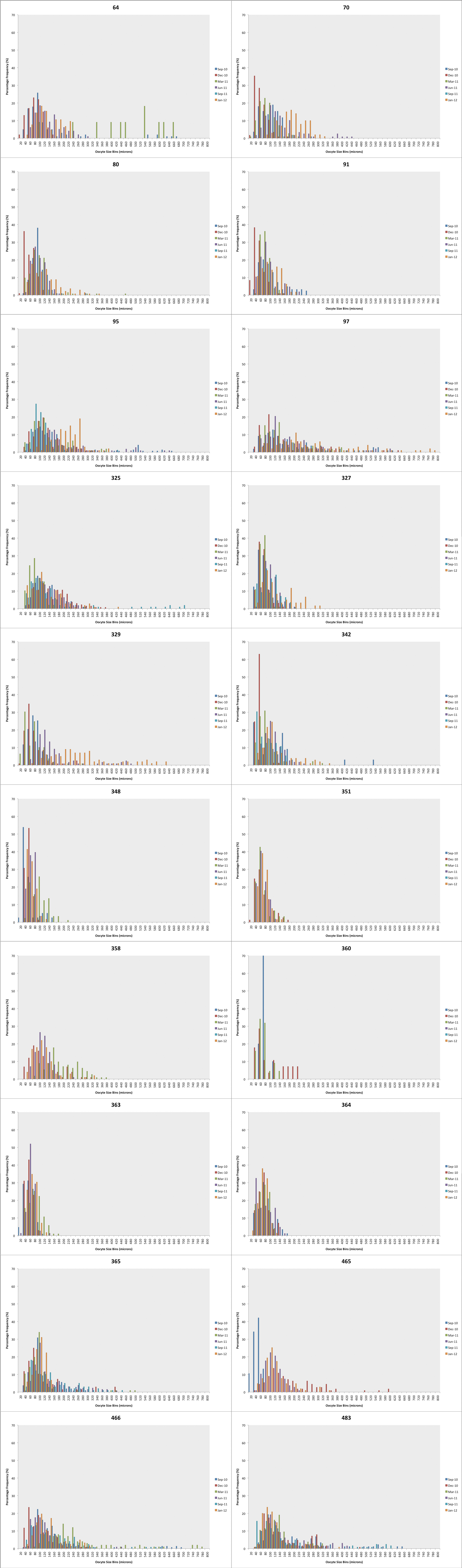

Supplement: Figure S2 — Graphs of individual oocyte size frequency data. Each graph is for a single individual with each month of data plotted. (TIF) [file pone.0090893.s002.tif]
